# Cytotoxic and Immunomodulatory Effects of Hypericin as a Photosensitizer in Photodynamic Therapy Used on Skin Cell Cultures

**DOI:** 10.3390/pharmaceutics16060696

**Published:** 2024-05-23

**Authors:** Magdalena Krupka-Olek, Andrzej Bożek, Zenon P. Czuba, Małgorzata Kłósek, Grzegorz Cieślar, Aleksandra Kawczyk-Krupka

**Affiliations:** 1 Doctoral School of the Medical University of Silesia, 40-055 Katowice, Poland; 2 Clinical Department of Internal Diseases and Geriatrics, Chair of Internal Diseases, Dermatology and Allergology in Zabrze, Medical University of Silesia, 40-055 Katowice, Poland; andrzej.bozek@sum.edu.pl; 3 Department of Microbiology and Immunology, Faculty of Medical Sciences in Zabrze, Medical University of Silesia, 40-055 Katowice, Poland; zczuba@sum.edu.pl (Z.P.C.); mklosek@sum.edu.pl (M.K.); 4 Department of Internal Diseases, Angiology and Physical Medicine, Center for Laser Diagnostics and Therapy, Faculty of Medical Sciences in Zabrze, Medical University of Silesia, 40-055 Katowice, Poland; cieslar1@tlen.pl

**Keywords:** photodynamic therapy, hypericin, skin cell cultures, immunomodulatory effect

## Abstract

Determination of the hypericin–photodynamic (HY–PDT) effect on the secretion of cytokines secreted by the skin cells, may be the basis for using the immunomodulatory effect of photodynamic action in the treatment of inflammatory skin diseases. The study aimed to evaluate the cytotoxic and immunomodulatory effects of hypericin (HY) in photodynamic therapy (PDT) performed in vitro on cultures of selected skin cell lines. The study used two human cell lines, primary dermal fibroblast (HDFa) and primary epidermal keratinocytes (HEKa). The MTT test was used to define the metabolic activity of treated cells. Cell supernatants subjected to sublethal PDT were assessed to determine the interleukins: IL-2, IL-8, IL-10, IL-11, IL-19, IL-22, and metalloproteinase 1 (MMP-1). The results confirm the destructive effect of HY–PDT and the immunomodulatory effects of sublethal doses on the selected skin cells, depending on the concentration of HY and the light doses. No statistically significant differences were noted in IL-2 and IL-10 concentration after HY–PDT for HEKa and HDFa lines. After using HY–PDT, the concentration of IL-8, MMP-1, IL-22, and IL-11 significantly decreased in the HEKa line. Moreover, the concentration of IL-19 and MMP-1 significantly decreased in the HDFa line. The concentration of IL-11 in the HDFa line after using only the HY, without the light, increased but decreased after HY–PDT. Our experiment confirmed that HY–PDT has not only a cytotoxic effect but, used in sublethal doses, also presents immunomodulatory properties. These may be an advantage of HY–PDT when used in the treatment of persistent skin inflammation, connected with the release of pro-inflammatory cytokines resistant to conventional treatment methods.

## 1. Introduction

**Figure 1:**
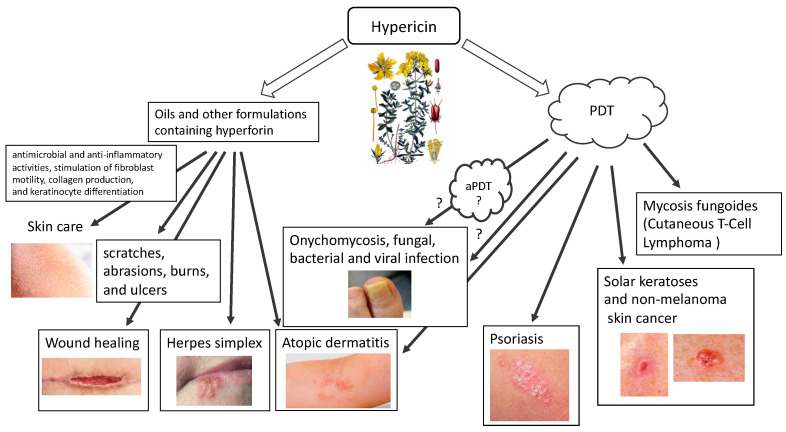
Use of hypericin in skin diseases (HY).

Hypericin (HY) (4,5,7,4′,5′,7′-hexahydroxy-2,2′-dimethylnaphthodianthrone) is a natural dye with hydrophobic properties found in some species of the genus Hypericum, especially *Hypericum perforatum* L. (St. John’s wort), basidiomycetes (*Dermocybe* spp.) and endophytic fungi (*Thielavia subthermophila*). As a natural substance with unexpectedly diverse and beneficial medical effects, it has been explored in recent decades for its wide pharmacological spectrum [1,2,3,4,5,6]. HY has antibacterial and antiviral, anticancer, antioxidant and neuroprotective properties, and has therefore been used in the treatment of many diseases, either as an independent substance or, using its photosensitizing properties, in photodynamic therapy [7,8]. *Hypericum perforatum* extracts have been used for thousands of years to treat abrasions, cuts and wounds [7] (Figure 1).

Oils containing hyperforin and hypericin or their derivatives are appropriate for application on scratches, abrasions, burns, and ulcers. The effectiveness of oil containing HY in the treatment is thanks to its antimicrobial and anti-inflammatory properties, which also follow from the stimulation of fibroblast motility, collagen production, and keratinocyte differentiation. The inflammation-reducing effects of HY are partly related to its antibacterial (e.g., directed against methicillin-resistant Staphylococcus aureus (MRSA) and penicillin-resistant (PRSA)) and antiviral (targeted against murine cytomegalovirus (MCMV), Sindbis virus, Friend’s virus, herpes virus and Ranscher virus). The advantage of HY is that it is highly photoreactive. Due to the relatively short distance between oxygen atoms (about 2.5 Å), hydroxyl hydrogen can transfer between hydroxyl oxygen and carbonyl oxygen in the presence of fluorescent light. The fluorescence spectrum of hypericin and its analogues shows a “protonated” carbonyl group, which supports hydrogen transfer and acidification of the surrounding environment. Therefore, hydrogen flows constantly between two oxygen atoms under the influence of fluorescent light. Having as many as two negative charges in its structure, it can diffuse through the plasma membrane and move in intracellular membranes. HY interacts with hydrophobic structures through non-covalent bonds such as hydrogen bonds, van der Waals forces, hydrophobic effects, and π-π stacking. Due to the balance between the anionic and hydrophobic nature in the compound structure, HY can localize to various parts of the cell, such as lysosomes and the Golgi apparatus, cytoplasm and endoplasmic reticulum.

**Figure 2:**
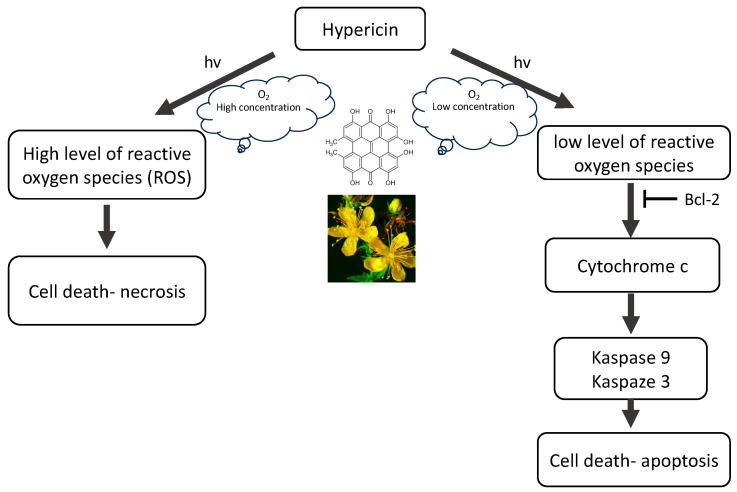
The mechanism of action of hypericin (HY).

Hypericin is known as a natural photosensitizing agent used in photodynamic therapy. In addition to its anticancer effect, it is characterized by fungicidal, bactericidal and antiviral properties [9,10,11,12,13,14,15,16,17,18,19,20,21]. HY’s optical properties configure its absorption of the visible light spectrum from 500 to 620 nm, with a maximum absorption at 595 nm, showing light emission of the order of 603 nm and is characterized by an intense red fluorescence [22]. The use of HY is made difficult by its phototoxicity, high temperature and pH, poor water solubility and high light sensitivity. The molar extinction coefficients of HY at approximately 590 nm range from 27,000 to 52,000 depending on the solvent, raw material, manufacturing process, aggregation, storage conditions, moisture, etc. The absorbance of the peak at 595 nm decreases upon increasing the pH, with the concomitant formation of a new, broad absorption band near 620–640 nm [23]. The quantum yield of singlet oxygen photosensitized by HY in air-saturated DMSO is Φ_Δ_ = 0.4 ± 0.03. The rate constant for HY triplet state depopulation in reaction with ground state molecular oxygen is k_q_ = 1.6 ± 0.3 × 10^9^ M^−1^ s^−1^. HY may act as a sensitizer in photodynamic reactions (mainly by type II mechanism). After activation with light, HY is effective primarily in the production of singlet oxygen (type II mechanism) but also superoxide anion (type I mechanism), which ultimately leads to necrosis, apoptosis, and autophagy, while, as an ICD type II inducer, HY–PDT can activate the immune system. There are two types of PDT reactions. Reactions that fall within the type I mechanism lead to the formation of radicals and radical ions that react with molecular oxygen, leading to the production of reactive oxygen species (ROS), such as superoxide anion, hydrogen peroxide (H_2_O_2_) and hydroxyl radical. In a second mechanism, the photosensitizer can transfer its energy to molecular oxygen via a triplet–triplet annihilation reaction, producing singlet oxygen (^1^O_2_). The production of ROS leads to lipid peroxidation and membrane damage, which is why hypericin and similar substances that sensitize to ^1^O_2_ are highly phototoxic. This type II reaction mechanism has been shown to predominate during PDT. However, it has been proven that the photosensitization reaction carried out by HY can be transferred from type II to type I and vice versa, using different concentrations of antioxidants [24]. Regardless of the initial reaction, both type I and type II mechanisms lead to oxidative damage and tumor destruction. The photocytotoxicity of hypericin is highly oxygen-dependent, resulting in the activation of various forms of cell death (Figure 2). Probably the main mechanism of action of HY–PDT is the release of mitochondrial cytochrome c and secondary impairment of mitochondrial respiratory functions. Nevertheless, the final effect of HY–PDT depends on many factors. It has been proven that the phototoxicity of hypericin depends on the light dose and its concentration, and it has also been shown that the location of the photosensitizer in the cell and the mechanisms of cell death (apoptosis and necrosis) depend on the cell type.

The anticancer effect of HY involves the induction of apoptosis by activating caspases, which are cysteine proteases that trigger a cascade of proteolytic cleavages in cells. In addition, HY also causes the release of cytochrome c from mitochondria, inhibiting the growth of cancerous tissues, including glioma, neuroblastoma, adenoma, mesothelioma, melanoma, carcinoma, sarcoma, and leukemia. Additionally, in the presence of light and oxygen, hypericin, acting as a natural photosensitizer, leads to the production of superoxide radicals, which in turn create superoxide or hydroxyl radicals, i.e., singlet oxygen molecules, with cytotoxic properties towards cancer cells. Furthermore, death signals other than the mitochondria-mediated caspase activation cascade contribute to the enhancement of hypericin-induced photokilling in some cell types. When using necrotic concentrations of HY, no activation of caspases is observed, despite the rapid release of mitochondrial cytochrome c into the cytosol, which is probably the result of direct inactivation of caspases as a result of oxidation of the cysteinyl residue, which in turn may block the apoptotic pathway and shift the balance towards necrosis. High levels of oxidative stress induced by necrotic PDT states can cause rapid and irreversible impairment of mitochondrial function, causing a decrease in ATP levels and inhibiting the possibility of cell death through apoptosis. Thus, HY–PDT is a potent inducer of the mitochondrial caspase activation pathway; however, the “extrinsic” caspase activation pathway may also contribute to cell photokilling. HY interferes with the release of anti-apoptotic Bcl-2 family members, including Bcl-2 and Bcl-XL, and inhibit cytochrome c efflux, while pro-apoptotic members such as Bid, Bax, and Bad promote its release. At the same time, immediately after irradiation, JNK1 and p38 MAPK are activated, while ERK are inhibited [1]. Clinical trials have shown that the use of HY–PDT has potential in the treatment of different neoplasmatic diseases, including recurrent mesothelioma and basal cell, and squamous cell carcinoma as well as psoriasis, warts and skin cancer malignant glioma, pituitary adenoma and cutaneous T-cell lymphoma [20,25,26,27,28].

There are various possibilities for using HY in skin diseases. Both in the form of traditionally used oils and in external application for the treatment of burns, including sunburns, wounds, blunt injuries, hemorrhoids, ulcers, bedsores, and colloidal scars [7]. In these skin diseases, the antibacterial, antifungal, antiviral, anti-inflammatory and antioxidant properties of hypericin are used. Hypericin also shows potential in the treatment of inflammatory skin diseases with autoimmune deficiencies, such as atopic dermatitis or psoriasis. Moreover, hypericin’s second form of use in the treatment of dermatological diseases is as a photodynamic therapy in the treatment of basal cell carcinoma of the skin, mycosis fungoides, and even as a potential treatment for melanoma. In a clinical trial, 8 patients with squamous cell carcinoma (SCC (40–100 µg) 2–4 weeks) and 11 patients with basal cell carcinoma (BCC (40–200 µg) 2–6 weeks) were treated with local application of intralesional hypericin and visible light irradiation 3–5 times a week [26]. Clinical remissions were observed after 6–8 weeks [26]. A clinical trial using HY–PDT in actinic keratosis (8 with AK), basal cell carcinoma (21 with BCC) and Bowen’s disease (5 with carcinoma in situ) (0.15–0.25% HY, 2 h, 75 J/cm^2^ red light) has also been described [29]. After this HY–PDT, authors described complete remission in 50% for AK, 28% in patients with superficial BCC, and 40% in patients with Bowen’s disease and partial remission in patients with nodular BCC.

Recently, the results of a multicenter, placebo-controlled, double-blind, randomized phase III clinical trial (FLASH study) conducted from December 2015 to November 2020 in medical centers in the USA using local application of 0.25% HY ointment in photodynamic therapy of patients were published in the early stage of mycosis fungoides (MF, chronic T cutaneous lymphoma (CTCL)). The study confirmed the effectiveness of such treatment, emphasizing its good safety profile and lack of mutagenic effect. The study population included 169 patients, and, after 6 weeks of treatment, HY–PDT was 49% more effective than placebo after 3 cycles. There were no serious drug-related side effects [30,31]. Additionally, Rook et al. confirmed, in a phase II placebo-controlled study, the effectiveness of photodynamic therapy with topical hypericin and visible light irradiation in the treatment of cutaneous T-cell lymphoma and psoriasis [32]. A new, undeniably interesting direction may be the use of antimicrobial PDT (aPDT) with HY, which is confirmed by research and the associated published results using HY–PDT f.e. in onychomycosis [33,34]. It has been observed that HY inhibits the proliferation of various tumor cells, including lung cancers, bladder, colon, glioma, breast, cervical, leukemia, hepatic, melanoma and lymphoma [20,21]. Its use in inflammatory skin diseases, with its immunomodulating effect, may allow this form of therapy in chronic inflammatory skin diseases that cannot be treated conventionally, such as psoriasis, atopic dermatitis, or skin infection [7,28,32,35].

Inflammatory skin diseases, such as atopic dermatitis or psoriasis, are always difficult medical problems. They are usually chronic and require various forms of therapy, and, unfortunately, some patients require lifelong treatment. Cytokines are involved in initiating and maintaining inflammation and are responsible for the chronic and recurrent nature of the disease [36,37]. When cytokines induce inflammation, macrophages activate their production. The newest group of drugs used to treat psoriasis and atopic dermatitis are substances that affect the immune system [38,39]. Anti-interleukin (IL) therapies are the primary treatment for patients with moderate-to-severe psoriasis, therefore an important topic would be the impact of photodynamic therapy on the release of interleukins that play a major role in the etiopathogenesis of psoriasis [40].

Inflammatory skin diseases, including atopic dermatitis and psoriasis, constitute an important clinical problem. The primary physical effective dermatology method for treating inflammatory skin diseases is phototherapy, including laser therapy, photodynamic therapy (PDT), bath-PUVA, and extracorporeal photochemotherapy [41,42]. It has been confirmed that photodynamic therapy has cytotoxic and immunomodulatory effects [43]. PDT targets inflammatory cells, interferes with the production of cytokines and has a significant antibacterial effect on atopic skin [44]. Therefore, it is important to determine the PDT effect on skin cells and their secretion of cytokines.

In recent years, the significant role of the immune system, including cytokines, in the etiopathogenesis and course of autoimmune inflammatory skin diseases has been demonstrated [45,46]. Determination of the immunological profile of patients with severe inflammatory skin diseases that are resistant to conventional treatment methods, such as atopic dermatitis and psoriasis, supported by in vitro studies of the effect of PDT on the cytokine production by cells of the skin, may be the basis for the possible qualification of this group of patients for photodynamic therapy. This would act through an immunomodulatory mechanism and would be minimally invasive, thus avoiding severe side effects. To test the hypothesis that HY–PDT has an immunoregulatory effect by influencing the secretion of cytokines from the keratinocytes and fibroblasts responsible for the inflammatory process, we examined the effect of HY–PDT on the immunological activity of keratinocytes and fibroblasts in culture, measuring the secretion of IL-2, IL-8, IL-10, IL-11, IL-19, IL-22, and MMP-1.

## 2. Materials and Methods

### 2.1. Chemicals

Dimethyl sulfoxide (DMSO), hydrocortisone, HY (Hypericin Calbiochem), and MTT (3-[4,5-dimethylthiazol-2-yl]-2,5-diphenyltetrazolium bromide) were acquired from Sigma–Aldrich (St. Louis, MO, USA). From ATCC (Manassas, VA, USA) provided DMEM: F-12, Dulbecc’s Modified Eagle’s Medium (DMEM), trypsin (0.23%)-ethylenediaminetetraacetic acid (EDTA) (0.53 mM), and inactivated fetal bovine serum (FBS). PAA Laboratories (Cölbe, Germany) supplied Dulbecc’s phosphate-buffered saline (DPBS) free of calcium and magnesium ions. The supplier of Bio-Plex Pro Assays was BIO-RAD Laboratories, Inc. (Hercules, CA, USA). All additional substances were pure or of analytical grade.

### 2.2. Cell Cultures

**Figure 3:**
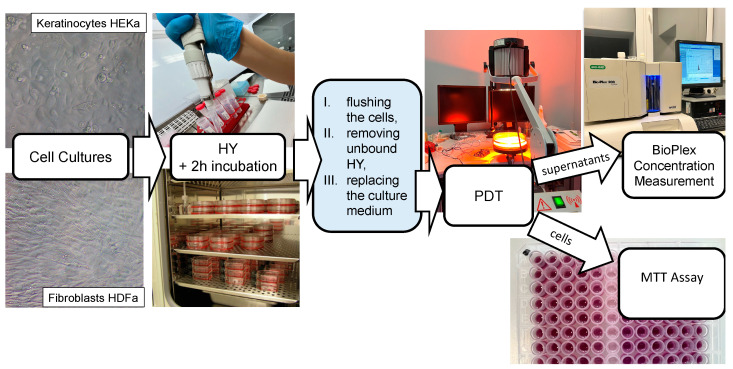
The scheme of the experiment HY–PDT.

The scheme of the HY–PDT experiment is presented on the Figure 3.

**Figure 4:**
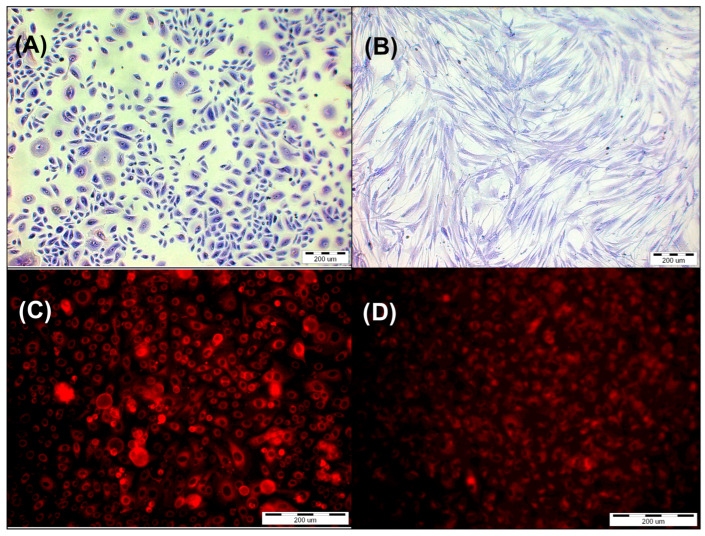
(**A**) Primary epidermal keratinocytes; normal, human, adult (HEKa) stained by Giemsa’s solution. (**B**) Primary dermal fibroblast (HDFa) stained by Giemsa’s solution. (**C**) HEKa line cells after incubation with HY in concentration 1 µM. (**D**) HDFa line cells after incubation with HY in concentration 1 µM.

The experiments were conducted on HEKa (Figure 4) primary epidermal keratinocytes (PCS-200-011) and HDFa (Figure 4) primary dermal fibroblasts (PCS-201-012). The supplier of cell lines was ATCC (Manassas, VA, USA). The cells HEKa were grown in monolayer cultures in dermal cell basal medium with 0.4% bovine pituitary extract (BPE), supplemented with rh TGF-α, L-glutamine, hydrocortisone hemisuccinate, rh insulin, epinephrine, apo-transferrin, 100 U/mL penicillin, 10 μg/mL streptomycin, and 25 ng/mL amphotericin B solution. The HDFa cells were grown in monolayer cultures in fibroblast basal medium supplemented with rh FGF-β, L-glutamine, ascorbic acid, hydrocortisone hemisuccinate, rh insulin, 2% bovine fetal serum, penicillin 100 U/mL, streptomycin 10 μg/mL, and amphotericin B solution 25 ng/mL. The cells were incubated in a humidified atmosphere at 37 °C with 5% CO_2_. The passages were conducted twice a week. The adhered HEKa and HDFa cells were treated with EDTA 0.02%, trypsin 0.05%, and trypsin neutralizing solution. The suspensions of 1.5 × 10^5^/mL HEKa cells and 1 × 10^5^/mL HDFa cells were used for the experiments. The next step of the study was to seed the skin cells in equivalent amounts of 200 µL per well into a 96-well plate. Incubation was then performed for 24 h to obtain adhesion.

### 2.3. Fluorescence Microscopy

The HEKa primary epidermal keratinocytes and HDFa primary dermal fibroblast adhered for 24 h in 96-well plates. The next day, the growth medium was removed, and the cells were washed with DPBS without calcium and magnesium ions. For two hours, HY was given to cells at the following final concentrations: 0.125 μM, 0.25 μM, 0.5 μM, and 1 μM. Dissolving HY in DMSO to a final concentration of ≤0.01% obtained the solution of 1 mM stock. After two hours, cells were rinsed with DPBS without calcium and magnesium ions, and the culture fresh medium was added. Using an Olympus IX51 inverted research microscope equipped with a color view camera (Tokyo, Japan, Olympus Inc.) and version 2.6 of Cell F software (Soft Imaging System GmbH, Münster, Germany) for capturing and analyzing images from microscope, the presence of HY in the cells was verified (Figure 4).

### 2.4. Photodynamic Therapy

In the next step of the research, following a 24 h period of adhesion, HEKa and HDFa cells were incubated for a 2 h period with HY at levels of 0 µM, 0.125 µM, 0.25 µM, 0.5 µM, and 1 µM. Since HY was added, the experiment was carried out without direct access to light. Following incubation of the cells with the PS, PBS without magnesium and calcium was flushed and replaced with the culture medium. Then, using an incoherent light source TO-1 (Cosmedico Medizintechnik Gm-bH, Schwenningen, Germany), the cells were exposed to visible light (VIS; 450–720 nm) as PDT (Figure 3). The infrared and orange light filters permitted the passage of wavelengths between 580–720 nm. For the experiment used the light doses of 1 J/cm^2^, 2 J/cm^2^, and 5 J/cm^2^ were used and the fluence rate was 35 mW/cm^2^. The distance between the light source and the plate containing the cells was 30 cm. To avoid the heating effect in the experiment a double water filter was used. Later, the cells were incubated for 24 h in the dark. The controls were cells without light exposure.

### 2.5. MTT Assay

The colorimetric MTT examination was performed to measure cell metabolic activity. MTT was used according to the typical protocol. Supernatants were gathered after 24 h of incubation and frozen at −80 °C, to preserve them for further experiment. The fresh medium with MTT was inserted into the cells at a concentration of 0.5 mg/mL and lasted four hours for incubation time. Subsequently, after incubation, the medium was injected with unreduced MTT, and DMSO was added in order to dissolve the formazan. The crystals were liquefied for ten minutes by shaking the plates after adding DMSO. After the transfer of the solution to a polypropylene plate, the absorbance was determined spectrophotometrically at a wavelength of 550 nm using a microplate reader (Bio-Tek Instruments Inc., eLx 800, Winooski, VT, USA). In the study, reduction of MTT was calculated as a percentage of the dark group control for each cell line.

### 2.6. Cytokines Measurements

In the next step of the experiment, IL-2, IL-8, IL-10, IL-11, IL-19, IL-22, and MMP-1 markings were undertaken with Bio-Plex Pro™ Assay kit and with Bio-Plex Suspension Array System (BIO-RAD Laboratories Inc., Hercules, CA, USA) (Figure 3). Assays were performed for control samples and samples treated with sublethal doses of HY–PDT at concentrations 0 µM, 0.25 µM, and 0.5 µM of HY. When we used the irradiation at a dose of 5 J/cm^2^, a decrease in the metabolic activity of cells measured by the MTT test was observed, which indicates the cytotoxic effect of HY PDT. Therefore, irradiation doses of 1 J/cm^2^ and 2 J/cm^2^ were considered sublethal and subsequent experiments aimed at measuring cytokines secreted by living cells were carried out only for doses of 1 J/cm^2^ and 2 J/cm^2^. After centrifugation, the supernatants were transferred to 96-well microplates and diluted with buffer to a volume of 50 µL. Standards of the analyses to be determined were also placed in the plate wells. Then, a suspension of magnetic beads coated with specific antibodies was added to the analyses. Further proceedings followed the manufacture’s instructions. The Bio-Plex Pro™ Assay kit consisted of 5.6 µm diameter fluorescent-dyed polystyrene beads that varied in color depending on the bound detection substance, a flow cytometer, and compatible software that automatically analyzed fluorescence results. After pre-wetting the well filter plate with wash buffer, the solution in each well was aspirated using a vacuum manifold. In the next step, the culture supernatants of the tested cells were incubated with antibody-conjugated beads for 30 min. Cytokines are determined using an antibody covalently bound to an appropriately colored polystyrene bead. After incubation, the test suspension was washed to remove unbound cytokine antibodies. In the next step, biotinylated specific detection antibodies that bind to the epitopes of the tested cytokines were added.

After another incubation and a series of washes, detection antibodies and streptavidin–phycoerythrin were added to the tested material for 30 min. Streptavidin has an affinity for the biotinylated detection antibody, and phycoerythrin is used as a fluorescent marker. After incubation, unbound streptavidin–PE was again removed from the solution by a series of buffer washes. The finally obtained beads covered with complexes labelled with phycoerythrin were analyzed in the Bio-Plex 3D Suspension Array System (Bio-Rad Laboratories Inc., USA). Beads associated with each cytokine were analyzed in a Bioplex Array Reader (Bio-Plex^®^ 200 system). The fluorescence intensity was assessed using the Bio-Plex Manager™ program and the concentrations of the tested cytokines corresponding to the fluorescence intensity were automatically calculated using this program. The concentrations of the determined cytokines were obtained based on standard curves using the manufacture’s software. The experiments were repeated four times (*n* = 4).

### 2.7. Statistical Analysis

The Shapiro–Wilk test was performed to check the normality of distribution. Regularity of distribution was gained in the case of the MTT test, and the proportion of MTT reduction of the examined unit concerning the control group was compared using the Student’s *t*-test. To calculate cytokine denseness, Kruskal–Wallis analysis of variance (ANOVA) with post hoc examination using the multiple comparison test was used to define cytokine concentrations. The statistical examination was used in Statistica version 13 (TIBCO Software Inc., Palo Alto, CA, USA, 2017), and the diagrams were arranged using Excel^®^ (Microsoft 365, 2303 version). Statistical significance was achieved for values of *p* < 0.05 (**) or *p* < 0.01 (***). The experiments were repeated four times (*n* = 4).

## 3. Results

### 3.1. Fluorescence Microscopy

HY uptake by both cell lines was observed by using a fluorescent microscope. The control groups that were not administered HY were shown to have no fluorescence. When HY concentrations in the culture medium increased, fluorescence in the HY-incubated cells increased as well (Figure 4).

### 3.2. MTT Cytotoxicity Assay

**Figure 5:**
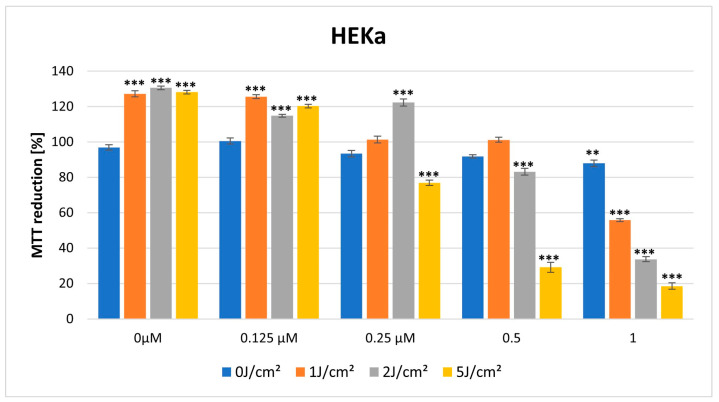
MTT reduction in the HEKa cell lines for different HY concentrations: 0 μM, 0.125 μM, 0.25 μM, 0.5 μM, and 1 μM and different light doses: 0 J/cm^2^, 1 J/cm^2^, 2 J/cm^2^, 5 J/cm^2^. The values represent the means ± SE. ** *p* < 0.05, *** *p* < 0.01.

**Figure 6:**
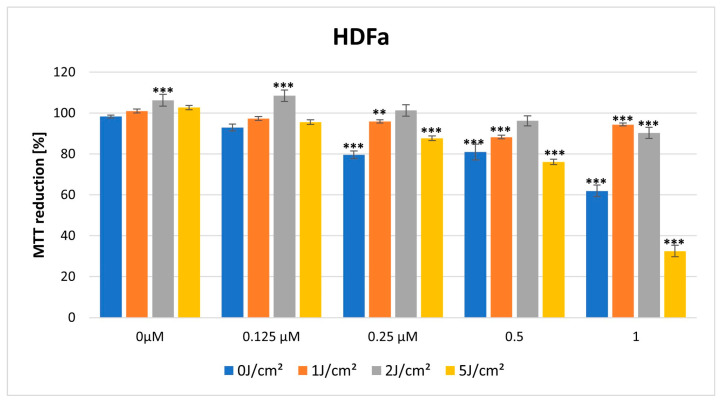
MTT reduction in the HDFa cell lines for different HY concentrations: 0 μM, 0.125 μM, 0.25 μM, 0.5 μM, and 1 μM and different light doses: 0 J/cm^2^, 1 J/cm^2^, 2 J/cm^2^, 5 J/cm^2^. The values represent the means ± SE. ** *p* < 0.05, *** *p* < 0.01.

In the dark, HY showed a cytotoxic effect on both lines in the MTT assay. The cytotoxic effect is more visible in the case of the HDFa cell line, with a significant reduction in MTT reduction noticeable at a dose of 0.25 μM HY (MTT reduction = 79.62% ± 1.77, *p* > 0.01), while in the case of the HEKa line, a statistically significant cytotoxic effect occurred for the highest dose of HY 1 μM (MTT reduction = 87.90% ± 1.80, *p* > 0.05) (Figure 5 and Figure 6).

For a light dose of 1 J/cm^2^, light-only therapy and also in combination with a low HY dose of 0.125 μM had a stimulating effect on HEKa line cells (MTT reduction = 127.18% ± 1.71, *** *p* < 0.01; MTT reduction = 125.63% ± 1.06, *** *p* < 0.01, respectively). In the case of HY concentration of 1 μM on this cell line, it already had a cytotoxic effect (MTT reduction = 55.77% ± 0.74, *** *p* < 0.01) (Figure 5 and Figure 6).

For a light dose of 2 J/cm^2^, a similarly light-only therapy, without HY and in combination with small doses of HY 0.125 μM, 0.25 μM had a stimulating effect on HEKa line cells (respectively, MTT reduction = 130.58% ± 1.03, *** *p* < 0.01; MTT 114.85% ± 0.84, *** *p* < 0.01; MTT reduction = 122.29 ± 1.94, *** *p* < 0.01). The cytotoxic effect in HEKa is noticeable at a 1 μM dose (MTT reduction = 33.71 ± 1.41, *** *p* < 0.01). In the HDFa line, a stimulating effect also appears in the concentration of HY 0.125 μM and light-only exposure (MTT reduction = 106.29 ± 1.68, *** *p* < 0.01, MTT reduction = 108.49 ± 1.22, *** *p* < 0.01). The cytotoxic effect in HDFa is visible in doses of HY 1μM (Figure 5 and Figure 6).

When a higher light dose of 5 J/cm^2^ was used, cytotoxicity significantly increased in both cell lines with increasing HY concentration (HEKa: MTT reduction = 18.55 ± 0.79, *** *p* < 0.01; HDFa: MTT reduction = 32.48 ± 2.42, *** *p* < 0.01) (Figure 5 and Figure 6).

### 3.3. Effect of HY–PDT on the Secretory Activity of IL-2, IL-10, MMP-1, IL-19, IL-22, IL-11, and IL-8

#### 3.3.1. IL-2

**Figure 7:**
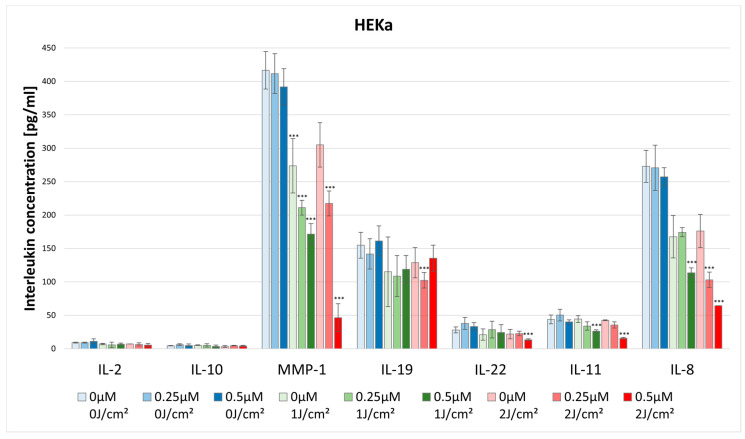
The concentration of IL-2, IL-10, MMP-1, IL-19, IL-22, IL-11, and IL-8 in the supernatants from cell culture HEKa line in different light doses (0 J/cm^2^, 1 J/cm^2^, 2 J/cm^2^) and HY concentrations (0 μM, 0.25 μM, 0.5 μM). Statistical differences were marked concerning the dark control group within a given cytokine. The values represent the means ± SD. *** *p* < 0.01.

**Figure 8:**
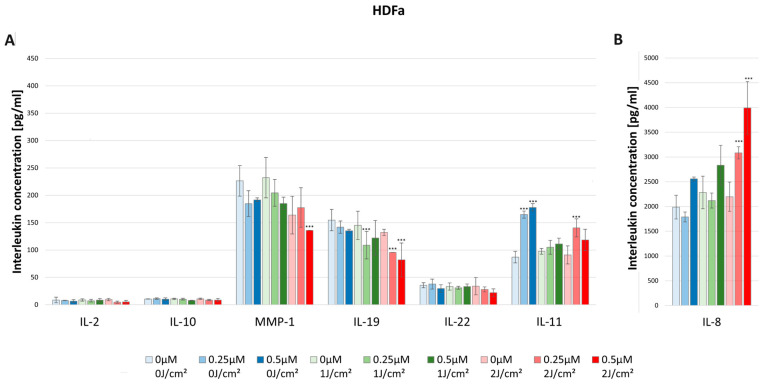
(**A**) The concentration of IL-2, IL-10, MMP-1, IL-19, IL-22, and IL-11 in the supernatants from cell culture HDFa line in different light doses (0 J/cm^2^, 1 J/cm^2^, 2 J/cm^2^) and HY concentrations (0 μM, 0.25 μM, 0.5 μM). Statistical differences were marked concerning the dark control group within a given cytokine. The values represent the means ± SD. *** *p* < 0.01. (**B**) The concentration of IL-8 in the supernatants from cell culture HDFa line in different light doses (0 J/cm^2^, 1 J/cm^2^, 2 J/cm^2^) and HY concentrations (0 μM, 0.25 μM, 0.5 μM). The values represent the means ± SD. *** *p* < 0.01.

No statistically significant differences were noted in IL-2 concentration after HY–PDT for both HEKa and HDFa lines. For the dark control without PS, in the case of HEKa cells, the IL-2 level was 9.01 pg/mL ± 0.70 pg/mL and in HDFa cells the level was 8.63 pg/mL ± 5.07 pg/mL (Figure 7 and Figure 8).

#### 3.3.2. IL-8

The concentration of IL-8 after using HY–PDT significantly decreased in the HEKa line. For the dark control the cytokine concentration was 272.96 pg/mL ± 23.91 pg/mL, and for the 0.5 μM at the light dose 1 J/cm^2^ the concentration significantly decreased to 113.64 pg/mL ± 7.30 pg/mL. Additionally, for the light dose of 2 J/cm^2^ the concentration dropped significantly at a dose of 0.25 μM HY to 103.19 pg/mL ± 11.29 pg/mL, and for the dose of 0.5 μM HY it decreased to 64.38 pg/mL (Figure 7).

In the case of the HDFa line, the concentration significantly increased after irradiation with a light dose of 2 J/cm^2^ in the concentrations of HY 0.25 μM (3084.52 pg/mL ± 123.60 pg/mL) and 0.5 μM (3991.77 pg/mL ± 530.95 pg/mL) compared with the dark control (1986.98 pg/mL ± 238.72 pg/mL). As one can see, the HDFa line produced much higher IL-8 concentrations than the HEKa line (Figure 8).

#### 3.3.3. IL-10

There were higher observed levels of IL-10 produced by HDFa than by HEKa cell line. For the dark control in the case of HDFa, the cytokine level was 10.60 pg/mL ± 0.39 pg/mL, while in the HEKa line it was 4.53 pg/mL ± 0.26 pg/mL. However, HY–PDT did not affect the levels of IL-10 secretion (Figure 7 and Figure 8).

#### 3.3.4. MMP-1

The concentration of MMP-1 after the use of HY–PDT significantly decreased in the HEKa line using the light dose of 1 J/cm^2^ with 0.25 μM (211.16 pg/mL ± 11.06 pg/mL) and 0.5 μM HY (171.50 pg/mL ± 15.67 pg/mL), and at the dose of 2 J/cm^2^ with 0.5 μM HY (46.41 pg/mL ± 20.89 pg/mL) compared with the control group (416.66 pg/mL ± 28.07 pg/mL). It is observable that, after using only the light dose of 1 J/cm^2^ with no HY, the concentration has decreased from 416.66 pg/mL ± 28.07 pg/mL to 273.70 pg/mL ± 40.55 pg/mL (Figure 7). Furthermore, the HEKa line produced significantly higher concentrations of MMP-1 than the HDFa line. In the case of the HDFa line, the statistically significant reduction of MMP-1 concentration was observed in the light dose of 2 J/cm^2^ with 0.5 μM HY (135.84 pg/mL ± 00.0 pg/mL) when compared with the control group (241.90 pg/mL ± 21.10 pg/mL) (Figure 8).

#### 3.3.5. IL-19

The concentration of IL-19 in HEKa line cells after HY–PDT significantly decreased in the light dose of 2 J/cm^2^ with a concentration of 0.5 μM HY (135.29 pg/mL ± 19.62 pg/mL) in comparison with the control group (154.82 pg/mL ± 19.45 pg/mL) (Figure 7).

In HDFa line cells, one can notice a decrease in the concentration of IL-19 at a light dose of 1 J/cm^2^ and a concentration of 0.25 μM HY (108.89 pg/mL ± 25.01 pg/mL) related to the dark control (154.82 pg/mL ± 19.45 pg/mL). In the case of a light dose of 2 J/cm^2^ a decrease in the concentration was also observed at the concentrations of 0.25 μM HY (95.70 pg/mL) and 0.5 μM HY (81.98 pg/mL ± 30.92 pg/mL) (Figure 8).

#### 3.3.6. IL-22

The concentration of IL-22 after the use of HY–PDT significantly decreased in the HEKa line during light irradiation of 2 J/cm^2^ and with a concentration of 0.5 μM HY (13.34 pg/mL ± 1.40 pg/mL) in comparison with the control group (28.09 pg/mL ± 4.55 pg/mL) (Figure 7). No statistically significant differences were noted in IL-22 concentration after HY–PDT for the HDFa line. For the dark control without PS, the level of IL-22 was 35.60 pg/mL ± 4.65 pg/mL (Figure 8).

#### 3.3.7. IL-11

The concentration of IL-11 significantly decreased in the HEKa line after using HY–PDT compared to the control group (43.82 pg/mL ± 6.70 pg/mL). After light irradiation of 1 J/cm^2^ and 2 J/cm^2^ in the concentration of 0.5 μM HY the level of IL-11 reduced respectively by 26.34 pg/mL ± 1.88 pg/mL; 15.88 pg/mL ± 1.33 pg/mL (Figure 7).

In contrast, in the HDFa line, the usage of only HY in doses of 0.25 μM (164.87 pg/mL ± 6.44 pg/mL) and 0.5 μM (177.67 pg/mL ± 2.52 pg/mL) increased the secretion of IL-11 compared to the control group of HDFa (87.07 pg/mL ± 10.47 pg/mL). However, after HY–PDT, the concentration decreased to the baseline. Additionally, at a light dose of 2 J/cm^2^ and 0.25 µM HY, the concentration of IL-11 increased again to 140.54 pg/mL ± 16.39 pg/mL (Figure 8).

## 4. Discussion

The idea of our study was to assess the impact of the PDT on skin cells, fibroblasts, and keratinocytes, to assess its immunomodulatory effect and possible possibilities of its use in inflammatory skin diseases such as atopic dermatitis (AD) and psoriasis. In our experiment, hypericin was chosen as the photosensitizer (PS), whose properties have previously attracted the attention of many researchers [31,32,33,34,35,36,37].

No such studies have examined the effects of HY–PDT on human skin cell types in an in vitro system; however, both in vitro experiments and clinical studies conducted with this natural photosensitizer indicate its effective and multidirectional effect.

Woźniak and Nowak-Perlak have confirmed that HY–PDT may be an effective method of treating patients with skin cancer, with the reservation that the use of hypericin is not easy due to disadvantages such as its poor solubility in water and sensitivity to heat and pH [47]. Nevertheless, we decided to choose this photosensitizer, taking into account its properties, natural origin, effectiveness and the possibility of safe use in inflammatory skin diseases not due to its cytotoxic effect but its immunomodulatory effect, which we wanted to demonstrate and prove in the experiment. In our experiment we used different doses of HY at 0.125 μM, 0.25 μM, 0.5 μM, and 1 μM for two hours as well. Then, after assessing the metabolic activity of cells using the MTT assay, doses of HY and light were selected, at which the cells showed viability comparable to the control. That was to examine the effect of the photodynamic reaction on the secretion of cytokines responsible for the inflammatory process in the skin by keratinocytes and fibroblasts. In this study, we reveal that small doses of HY, 0.125 μM and 0.25 μM, and light, 1 J/cm^2^ and 2 J/cm^2^, stimulate the metabolic activity of cells measured by the MTT test. In contrast, higher concentrations of HY and light doses (0.5 μM HY and 5 J/cm^2^) have an inhibitory effect, both in the study on fibroblast (HDFa) and keratinocyte (HEKa) lines, confirming the cytotoxic effect, dependent on the photosensitizer dose and light, previously demonstrated in the work by Woźniak.

Popovic et al. also experimented to assess HY–PDT effects in vitro on the skin cell types. In a similar manner to our experiment, normal primary human keratinocytes, melanocytes, and fibroblasts were chosen in the study, which represented both the epidermal and dermal cellular compartments of human skin. The authors prepared an HY extract in dimethyl sulfoxide to obtain a 2 mM stock solution. Cells were incubated for four hours in different solutions in the range of 0.25–4 μM, and then HY was triggered with a diode-pumped laser at 561 nm light in the power density of 20 mW and a fluence rate of 5 J/cm^2^. The use of 3 µM HY–PDT (four hours incubation period, tunable laser 561 nm, 5 J/cm^2^, 20 mW) was cytotoxic to Fb and Mc, but not to Kc, and showed that lower doses of HY (0.25 μM, 0.5 μM, 0.75 μM, 1 μM, 2 μM) result in changes in cell morphology and thus affect various cellular functions. However, the authors discovered differences in the in vitro model between the susceptibility of primary cultured human skin cells to HY–PDT. They found that 3 μM HY–PDT is cytotoxic to fibroblasts to a minor extent in melanocytes but completely obstinate to keratinocytes. Additionally, the authors reveal that significant differences exist in cell viability and morphology between the cells [48]. Therefore, in our experiment, we examined not only the effect of cytotoxic doses of HY–PDT on fibroblasts and keratinocytes but, furthermore, the immunomodulatory activity of photodynamic action using hypericin for its possible use in skin inflammations that are characterized by the high production of cytokines involved in psoriasis and atopic dermatitis. One of the main cytokines involved in atopic dermatitis (AD) and psoriasis is interleukin-2 (IL-2). This cytokine has essential roles in key functions of the immune system, tolerance, and immunity, primarily via its direct effects on T cells [49]. IL-2, depending on its concentration, may exert both immunostimulatory and immuno-suppressive effects in autoimmune diseases, because, at high doses, IL-2 activates the differentiation and expansion of effector and memory T cells, while, at low doses, IL-2 activates the differentiation, survival and function of regulatory T cells (Tregs), a subset of CD4+ T cells [50]. In our experiment, we noted no statistically significant differences in IL-2, which may be related to the dose of photosensitizer, and energy dose, and may also have some beneficial clinical implications in terms of not triggering a possible controversial or unfavorable effect of HY–PDT on the release of this cytokine.

Another important cytokine for AD and psoriasis is interleukin 8. IL-8 levels are increased in psoriatic scale extracts and suction blister fluids in psoriatic skin lesions [51]. In our experiment, although we did not note the effect of HY–PDT on IL-2 secretion, the concentration of IL-8 significantly decreased in the HEKa line, which, considering the role of this cytokine in AD and psoriasis, seems to be an extremely beneficial effect. In our study we noted no statistically significant differences in IL-10 concentration after HY–PDT for HEKa and HDFa lines, which would mean no effect on anti-inflammatory cytokines; however, the lack of inhibition of the secretion of this interleukin, obtained in our experiment, can be taken into account as a beneficial factor.

In a study conducted by Larisch et al., the authors showed that a low dose of HY– PDT affects the secretion of cytokines tested on three human cell lines: HaCat cells, immortalized dermal keratinocytes, A431 cells epidermoid carcinoma cell line (keratinocytes from squamous cell carcinoma), and a primary dermal fibroblast cell line. The authors demonstrated that HY–PDT reduces IL-6 levels in all cell lines. Depending on the conditions, HY–PDT can cause an increase in IL-6 in malignant cells, which induces a specific anti-tumor immune response. The authors showed that, after treatment with HY–PDT, the level of IL-6 in A431 cells in both inflammatory conditions becomes significantly reduced when using a wavelength of 435 nm, while irradiation using a red wavelength of 610 nm had an IL-6-stimulating effect. Moreover, research has confirmed that hypericin itself can increase the level of anti-inflammatory cytokines IL-4, -5 and -10, which is extremely important in terms of its anti-inflammatory effect, alleviating inflammation in skin diseases or accelerating wound healing [52,53]. The effect of HY on its own, without photodynamic action, on the secretory activity of cells observed by these authors was also confirmed in our experiment.

In our study, we used an incoherent light source that enabled 580–720 nm wavelengths to flow through orange and infrared light filters. The concentration of IL-8, MMP-1, IL-22, and IL-11 significantly decreased in the HEKa line. Moreover, the concentration of IL-19 and MMP-1 significantly decreased in the HDFa line. This may confirm the results of Larisch et al. that the wavelength has an impact on the reduction of pro-inflammatory cytokines tested in our experiment, such as IL-8, MMP-1, IL-22, L-11 in the HEKa line and IL-19 and MMP-1 in the HDFa line. Moreover, we observed that the concentration of IL-19 and MMP-1 significantly decreased in the HDFa line. The concentration of IL-11 in the HDFa line after using only the HY, without the light, increased; however, after HY–PDT, it decreased, which would indicate not only the photodynamic effect but also the strong properties of hypericin itself. Larisch et al. have also noted a similar situation, though in the case of IL-10 expression in A431 cells, where hypericin itself caused an increase in this interleukin, and where this effect was intensified after lipopolysaccharide (LPS) induction and irradiation. This is an extremely desirable effect, given that IL-10 is one of the most important anti-inflammatory cytokines, and is involved in infections, acute and chronic inflammation and autoimmune disorders, particularly psoriasis and atopic dermatitis [52].

IL-22 plays an important role in psoriasis, atopic dermatitis, scleroderma and contact dermatitis. Furthermore, the PASI score, which measures the severity of the disease, is significantly linked with IL-22 plasma levels, suggesting that this interleukin may have a role in the etiology of psoriasis [54,55]. In our research, we showed that IL-22 significantly decreased in the HEKa line. Considering the important role of this cytokine in the skin’s defense process, this is, unfortunately, not a very beneficial or expected effect. Nevertheless, considering the dependence of the photodynamic effect on the type of photosensitizer, energy dose and research model, it can be hoped that this effect will not be confirmed in clinical trials. In our experiment we reveal that, after HY–PDT, the concentration of IL-11 significantly decreased in the HEKa line, which seems to be a favourable result from the point of view of the unfavorable and excessive effect of fibrosis, often occurring after photodynamic therapy during the healing phase. IL-11 has also been shown to stimulate the release of the alarm cytokine IL-33 in fibroblasts, triggering an inflammatory response in the local microenvironment. Unfortunately, such long-term stimulation ultimately leads to fibrosis, playing an important role in diseases such as systemic sclerosis (SSc) [56,57]. Extensive clinical trials remain necessary to confirm the efficacy of rhIL-11 as an anti-inflammatory agent in psoriasis patients [46].

In our study, we reveal that MMP-1 significantly decreased in the HDFa line, in terms of its antifibrogenic effect, the effect we obtained in terms of the benefits of the healing process after photodynamic therapy may be questionable. Metalloproteinases play a very important role in both physiological and pathological conditions of the body. They take part in the process of wound healing, and scar formation, they also participate in the processes of angiogenesis and apoptosis, autoimmune and inflammatory diseases such as psoriasis, atopic dermatitis and scleroderma. MMP-1 may be a potentially effective antifibrogenic agent for the prevention or treatment of hypertrophic scars [58]. In our study, the concentration of IL-19 significantly decreased in the HDFa line and, considering the role of this cytokine in atopic dermatitis, this seems to be an extremely beneficial phenomenon. The concentration of IL-19 in HEKa line cells, after using HY–PDT, significantly decreased in the light dose of 2 J/cm^2^ with a concentration of 0.5 μM HY, in comparison with the control group. In HDFa line cells one can notice a decrease in the concentration of IL-19 at a light dose of 1 J/cm^2^ and a concentration of 0.25 μM HY related to the dark control. This anti-inflammatory effect of photodynamic therapy is extremely desirable in inflammatory skin diseases. It has been shown that, in atopic dermatitis, IL-19 is highly expressed in skin lesions [59]. IL-19 shows the most robust differential expression between psoriatic lesions and healthy skin. Cutaneous IL-19 overproduction is reflected by elevated IL-19 blood levels that correlate with psoriasis severity [60].

We simultaneously conducted a clinical trial in our center, which included 139 people suffering from AD, 115 with PS and a control group of 142. Of all of these people, cytokine profiles were determined in the blood serum for IFN-γ, IL-4, IL-2, IL-6, IL-5, IL-12, IL-8, IL-17, TNF-α, IL-22, IL-18, and IL-24 in ELISA. The examination of the above cytokines showed their high similarity in the examined individuals and validated the fact that AD and PS are active inflammatory diseases. Therefore, the next step in our investigations was to determine the effect of HY–PDT on cytokine secretion in vitro studies, in order to use HY–PDT in AD and PS patients [61]. Of course, the complex nature of the mechanism of action of cytokines and their mutual relations are extremely complicated; however, it seems that HY–PDT, which is a natural substance with multidirectional action, may find its place in the treatment of persistent inflammatory skin diseases, such as atopic dermatitis or psoriasis, that cannot be treated with conventional methods.

HY itself also has very interesting effects. This substance inhibits the breakdown of serotonin, a neurotransmitter also called the “happiness hormone”, and is also an inhibitor of the reuptake of serotonin, norepinephrine, and dopamine. Classic antidepressants work similarly. Studies have shown that, for patients with mild-to-moderate depression, which has also been shown in AD and PS patients, St. John’s wort has comparable effectiveness and safety to SSRIs. All of these reports are extremely interesting and indicate the great potential of HY–PDT in inflammatory skin diseases, not only due to the immunomodulatory effect of HY–PDT, but also the interesting properties of HY itself, which confirms the sense of our research and the possibility of using it in a clinical trial.

## 5. Conclusions

Hypericin is an effective and natural photosensitizer that can be used in the PDT of inflammatory skin diseases that do not respond to conventional therapy. There are still many cases of patients that require additional forms of therapy to alleviate the course of this disease. HY–PDT may be such an option, as a minimally invasive method based on a natural photosensitizer. Determining the cytokine profile in patients suffering from atopic dermatitis and psoriasis and assessing the effect of HY–PDT on these proteins seems advisable to determine the effectiveness and sense of using this form of therapy and its safety. Additional fundamental and clinical research remains necessary to fully elucidate the roles of various interleukin proteins in the pathogenesis of inflammatory dermatologic diseases and HY–PDT outcomes in patients.

## Data Availability

Data are available on request.
